# Promoting coping competence for psychological stressors in nursing training: a controlled pedagogical intervention

**DOI:** 10.3389/fmed.2024.1429541

**Published:** 2024-09-27

**Authors:** Julia Warwas, Philine Krebs, Wiebke Vorpahl, Ulrike Weyland, Larissa Wilczek, Susan Seeber, Eveline Wittmann

**Affiliations:** ^1^University of Hohenheim, Chair of Business Education, especially Theory and Didactics of Vocational Education, Stuttgart, Germany; ^2^University of Göttingen, Chair of Business Education and Human Resource Development, Göttingen, Germany; ^3^University of Münster, Institute of Educational Science with a Focus on Vocational Education, Münster, Germany; ^4^Technical University of Munich, Department of Educational Sciences, Munich, Bavaria, Germany

**Keywords:** occupational stress, coping skills, pedagogical intervention, competence gains, evaluation, nursing training

## Abstract

**Background:**

Multiple stressors as well as health-and quality-impairing effects of strain in the nursing profession require the systematic acquisition of competence in dealing with these demands, starting at the stage of initial vocational training. This study investigates whether an instructional design, which merges didactic principles of nursing education with concepts and training measures from stress psychology, promotes the acquisition of stress coping competence more effectively than regular teaching on the relevant curricular field at nursing schools.

**Methods:**

The quasi-experimental study design, based on the Solomon four-group plan, included 332 trainees in Germany. The assessment of stress coping competence at the beginning and at the end of the intervention provided a video-stimulated situational judgment test covering nursing-specific stressful situations. All were validated by field experts. Complementing group comparisons, regression analyses examined intervention effects at the individual level while controlling for other predictors of learning success.

**Results:**

The highest solution rates for the two basic dimensions of stress coping competence, i.e., (1) *situation assessment* and (2) *strategy selection and justification*, occurred in the treatment classes without a pretest. At the individual level, treatment effects were confirmed for the first dimension. Students with a migration background showed lower competence gains than other students.

**Conclusion:**

The instructional design and the competence test provide valuable foundations for promoting and for diagnosing coping skills. Nevertheless, subsequent studies should examine adaptive support for different learner groups. Furthermore, additional observational phases should focus on the deliberate practice of acquired coping strategies in the practical training settings of nursing education.

## 1 Introduction

The ability to cope productively with stress and strain in the nursing profession is highly relevant in two ways (1, 2). From an individual perspective, *stress coping competence* constitutes a prerequisite for long-term commitment to and satisfaction with the profession, since chronic forms of occupational stress contribute to dissatisfaction, psychosomatic disorders, physical complaints and burnout among nurses (3). From a societal perspective, it contributes to maintaining a skilled workforce and a functioning health care system. High levels of stress reduce the quality of care provided by the affected nurses, including reduced empathy with care recipients and higher error rates in the care process (2). However, the design of effective pedagogical interventions to improve *stress coping competence* is challenging, given the multitude of daily stressors in the nursing profession, ranging from patient resistance against care measures to tight work schedules and (inter-)professional tensions (4, 5). Their impact on stress levels reaches a critical peak under conditions such as pandemic outbreaks and a systemic increase in the need for care within an aging society (6). There are many attempts to integrate issues such as health awareness and coping strategies into nursing education curricula. The current German curriculum for general nursing training can serve as an example of this trend, as it includes the protection of one’s own health as a discrete competency objective for nurses [(7), “Act on Nursing Professions”; (8), “Training and Examination Regulations for the Nursing Professions”]. However, systematic approaches to competence development and valid assessments of the effects of stress training have only increased in recent years (9, 10).

In this paper, we discuss the design and results of an intervention study aimed specifically at promoting *stress coping competence* among prospective nurses in an advanced stage of their professional qualification. In Germany, nursing education does not follow a predominantly academic approach, but is a combination of vocational school and practical training in nursing facilities. It usually lasts 3 years. The intervention consisted of a 12-hour instructional unit to be delivered during school hours. With a terminological and conceptual basis in work and stress psychology, it combines common and evaluated topics from stress management training, adapted to the working contexts of the target group, with didactic elements from nursing education in the German vocational system. This way, a scientifically based and nursing-specific intervention was created for application in the school setting of nursing education, following an action-oriented, situational approach to instructional design as the leading approach in this setting.

Our study investigates whether and to what extent this pedagogical intervention promotes the development of *stress coping competence*. To this end, a quasi-experimental design employing the Solomon four-group plan (11) (31) was implemented. This approach involves a total of two experimental groups and two control groups, but only one of the experimental groups and one of the control groups is tested *prior to* the intervention for the target variable of the treatment. Thus, it accounts for the effects of time-related confounding variables and allows a more robust estimation of a net treatment effect. The data set includes a sample of 332 trainees. To measure the participants’ levels of *stress coping competence*, a nursing-specific, empirically validated and digitally supported test instrument was applied (12, 13).

## 2 Design of the instructional unit to promote stress coping competence

### 2.1 Scientific foundations

Occupational science distinguishes stressors from stress reactions. While stressors describe all potentially harmful influences residing in a person’s work environment, stress reactions are the short-term, still reversible psychological or (pscho-)somatic consequences of these stressors within the person (14). Stressors and stress reactions are shaped by work tasks as well as proximal and organizational working conditions (15). Accordingly, task-immanent stressors of care work, such as being confronted with suffering and dying, are often accompanied by impediments to professional action regulation, such as the concurrence of different care needs for different patients (proximal working conditions), and are also exacerbated by an understaffing in care facilities (organizational conditions).

*Coping* involves the cognitive and behavioral efforts to deal with external and/or internal demands, such as the stressors mentioned above in a working context, which are weighed against accessible resources [(16), p. 141]. Whenever demands are appraised as exceeding resources, coping efforts set in. Drawing on evaluated stress management trainings (17–19), the instructional unit that was developed for prospective nurses classifies these efforts into three approaches:

- *Instrumental coping* entails measures to reduce the most prevalent stressors, such as eliminating coordination problems within a nursing team. It also entails measures to strengthen resources, such as optimizing individual work routines. Thus, this approach aims to actively change the stress-inducing or stress-reducing constituents of a work situation, thereby helping to regain individual capabilities to regulate professional actions.
- *Mental or cognitive coping* encompasses strategies to modify subjective perceptions and evaluations of the stressors inherent in working tasks or conditions. Accordingly, methods such as cognitive restructuring (i.e., re-appraisals) are associated with this second approach.
- *Palliative-regenerative coping* focuses on alleviating the experienced stress reaction rather than changing the situation-specific stressors *per se* (instrumentally). It therefore aims to reduce acute negative psychosomatic effects through emotion regulation, relaxation techniques and moments of recovery within or after the stressful situation.

### 2.2 Stress coping competence as the target variable

Against this general theoretical background, *stress coping competence* refers to individual knowledge and skills for dealing productively with domain-specific stressors and for preventing or regulating stress reactions in the work of professionals, with the aim of maintaining one’s own mental health (20). This particular competence is the learning objective of the developed instructional unit. It is well aligned well with an established, subject-centered notion of competence for professional work in the sense of individual prerequisites for action (21), which are themselves “acquired or expanded in self-organized, subject-oriented educational processes” (7) (“Act on Nursing Professions”). In work contexts, competence as a latent construct always includes a bundle of complementary facets that are relevant for dealing with professional demands (e.g., professional knowledge, skills, and attitudes for planning and implementing care measures). With these individual prerequisites, highly competent persons are able to make responsible, appropriate decisions and act effectively in a variety of situations in their professional domain [(22), p. 307]. Successful mastery of different situational demands (i.e., individual performance) allows observers to draw diagnostic conclusions about existing or lacking knowledge, skills, etc. (i.e., the individual level of competence).

**Figure 1:**
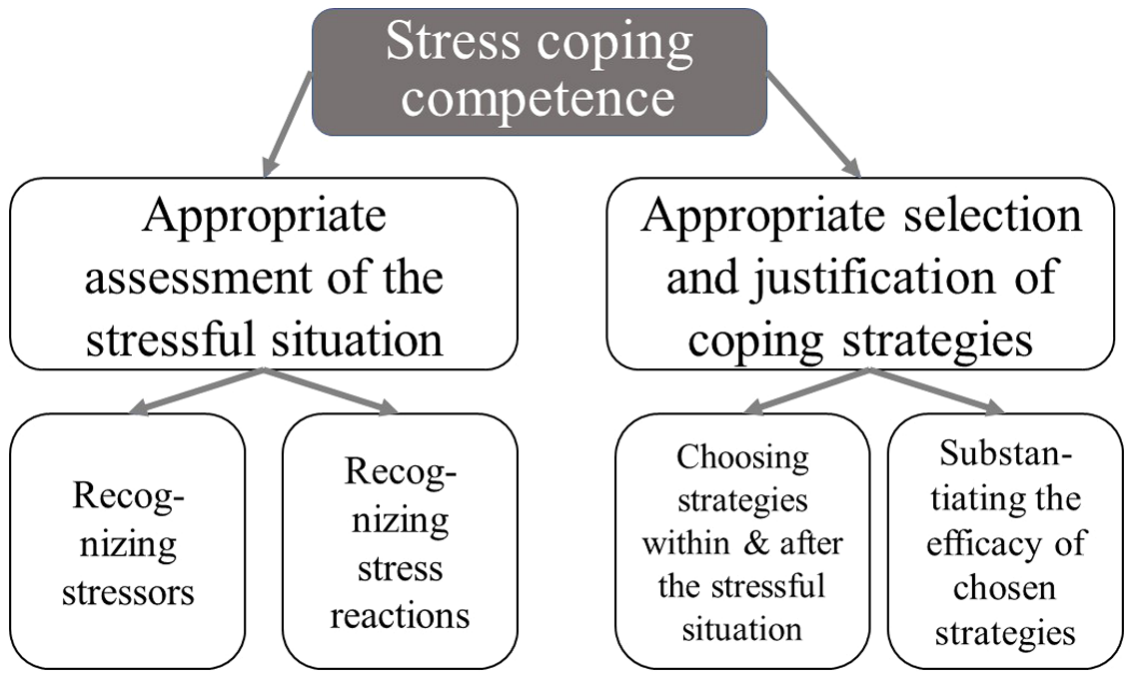
Proposed dimensional structure of stress coping competence.

*Stress coping competence* in occupational settings comprises a person’s ability to *discern the constituents* of a potentially distressing work situation as a first important facet. This ability permits an accurate evaluation of which stressors are present in a given situation and whether these factors can or cannot be modified (instrumentally). This ability represents an indispensable prerequisite for effective counteraction, as the nature of stressors markedly influences the range of situationally appropriate strategies, i.e., coping measures that can be executed successfully under given work requirements and circumstances. In addition, the suitability of a coping strategy depends on the scope of one’s behavioral repertoire and the accessibility of sources of support within the working environment, i.e., external resources that can be mobilized. Therefore, *coping flexibility* plays an important role in *strategy selection and implementation*, which delineates the second facet of *stress coping competence*. Coping flexibility refers to a person’s ability to variably and effectively modify coping behaviors according to the limiting and enabling features of the stressful situation at hand (23). This adaptive process typically incorporates strategies with complementary functions (instrumental, mental, and palliative-regenerative) and disparate timeframes of implementation (immediate and long-term). It distinguishes the concept of *coping competence* from the concept of *coping styles*, which postulates preferred, habitualized ways of coping regardless of varying situational characteristics (13, 24). Figure 1 provides an overview of the dimensionality of this psychological construct.

### 2.3 Instructional design of the intervention

To establish the essential features of the instructional unit, the manuals of evaluated stress management trainings for occupational settings were examined with regard to their structure and organization (schedule, group size, etc.), content (functional types and specific forms of coping strategies) and effectiveness (indicators of well-being and health). Furthermore, curricular analyses for the school-based phases of nursing training served to identify the optimal thematic and temporal context for implementing a coping-related pedagogical intervention. The curricular field 4 *“Promoting health and acting preventively*” for the vocational qualification of nursing specialists, which has mandatory status in the whole country today, provides ample opportunities for this purpose. The developmental process concluded with two sequential workshops together with teachers. These workshops sought to optimize the instructional unit from the application-oriented perspective of nursing didactics and to tailor it to the target group (trainees rather than experienced nurses). In the initial workshop, seven teachers, serving as faculty representatives within their schools, discussed the fundamental concept of the instructional unit. They made refinements to structure, utilization of media and the overall mix of instructional methods. The subsequent workshop concentrated on the specific implementation issues of singular lessons. It involved nearly 20 teachers who tackled this task in a division of labor. A consolidation phase aimed to maximize consistency in teaching.

The workshops yielded lesson plans for 12 discrete thematic sections, each comprising 45 min of instructional time.^1^ In accordance with a fundamental didactic tenet of the German system of vocational education and training, the lessons are designed to be action-oriented and situation-centered (25, 26). A radio play or video vignette serves as an anchor for all subsequent steps of skill acquisition, providing a vivid, complex and authentic scene within a nursing setting, which may be a nursing home for the elderly. This anchoring approach allows the learners to empathize with a protagonist who faces prototypical demands and challenges as a novice nurse. He/she has to take responsible, deliberate actions in order to deal effectively with the presented stress factors (e.g., time pressure and multiple care tasks). Following the didactic principle of action-orientation, students continually receive strategic impulses throughout the lessons to construct, revise and evaluate a multi-facetted action plan for dealing with the causes and impeding consequences (i.e., stressors and stress reactions) of the anchoring scene. The latter contains various types of stressors, thus paving the way for a coping attempt that combines several strategic elements (see section 2.1).

- *Stage 1—Informing and Planning.* At the prompting of their teacher, students begin to collect and intuitively assess a wider range of possible approaches for coping with the scenario-embedded demands. Thus, they draft a provisional action plan encompassing a range of strategic options.
- *Stage 2—Thematic Deepening and Practical Exploration.* The students examine and practice exemplary techniques for implementing diverse strategic approaches. During this investigative process, they thoroughly reflect the functional value of particular coping strategies for reducing stressors and regulating stress reactions. In light of the fact that the subjective appraisal of a situation is the determining factor in whether it is perceived as stressful, threatening, or even loss-making, the students initially focus on *mental coping strategies*. Specific techniques, such as cognitive restructuring and the creation of relieving or defusing thought patterns, are examined in detail. Subsequently, the students are guided by their instructor to develop an *instrumental approach to coping*, which allows for the immediate or long-term modification of specific stressors. In alignment with this methodology, the instructional unit introduces students to targeted, instrumental techniques for analyzing and prioritizing individual task assignments, as well as problem-solving heuristics. The final step in the investigative process is to reduce negative stress reactions. The students are instructed that strategies derived from the *palliative-regenerative approach to coping* are applicable in situations where the stress-inducing aspects of their work tasks or environments are unalterable or when supplementary strategies are sought to reduce the experienced strain. Consequently, they investigate techniques for emotion regulation, relaxation, and recovery within and after a stressful situation.
- *Stage 3—Option Selection and Decision-Making.* All potential strategic options are evaluated in comparison to one another in order to facilitate the selection of a reasonable and deliberate course of action. Moreover, students examine the suitability and applicability of these strategies in the authentic anchoring scenario, thereby enhancing their awareness of *coping flexibility*. Given the multifaceted nature of the authentic scenario, students are motivated to pursue a coping strategy that incorporates a combination of strategic elements (see section 2.1).
- *Stage 4—Integration and Control.* Students are required to consolidate the strategies they have identified as being the most effective for managing stressors and regulating stress reactions, as depicted in the anchoring scene, into a comprehensive, detailed, and testable action plan.
- *Stage 5—Summative Evaluation.* The class members evaluate their plans collectively under the guidance of the instructor and reflect on their learning progress and achievements.

In terms of methodological elements, the instructional unit employs a variety of social forms, including individual, partner, and group work. Moreover, a collection of explanatory videos and interactive worksheets is available for reference. A digital knowledge quiz may be used to assess the learner’s comprehension of the material presented. These design elements, in conjunction with comprehensive lesson plans, are made available to educators as *open educational resources* (https://ekge-lle.uni-goettingen.de/).

## 3 Materials and methods for evaluation

### 3.1 Aims of the study

The reported evaluation study sought to examine

1. whether *nursing classes* at vocational schools in Germany, which received a pedagogical intervention in the form of the newly developed instructional unit, demonstrated higher gains in *stress coping competence* than nursing classes receiving traditional instruction.
2. whether *individual competence gains* can be attributed to participating in the instructional unit rather than to individual learning prerequisites of each student.

These questions are investigated while controlling for pretest effects at the *class level* of analysis by employing a four-group-design (see next section). At the *individual level*, the predictive value of the individual group assignment was estimated together with other characteristics of a participant that might affect their learning process and success. These included prior knowledge, enrollment in a particular training program of nursing education, and basic biographical data. Competence gains were assessed in a validated test environment with authentic, video-based stimuli [see (13), for details]. For the scaling of measurement data, psychometric models were used, in particular probabilistic test models. These models facilitate the integration of task requirements and competence levels on a unified scale, while also enabling a more nuanced examination of the construct’s dimensionality (13).

### 3.2 Overview of the Solomon four-group plan

As an extension of the classic pre-post design, Solomon’s four-group plan comprises the following four sample groups, as depicted in Table 1 (11): Group 1 (pretest, treatment, posttest; hereafter PTP) and Group 2 (no pretest, treatment, posttest: -TP) represent the experimental groups, which differ only regarding the completion of a pretest. The third group (pretest, no treatment, posttest: P–P) takes both a pre-and posttest without taking part in the intervention and, thus, resembles a classic control group, while the fourth group (no pretest, no treatment, posttest: --P) only receives the posttest. For the present study, it should be noted that the control groups received regular classroom instruction on stress and strain (instead of the targeted instructional unit described in section 2), as all participating classes were required to cover the mandatory curricular area of “Health Promotion and Prevention” in nursing education.

**Table 1:** Solomon four-group plan.

Sample group	Pretest	Treatment	Posttest
Group 1 (PTP)	X	X	X
Group 2 (-TP)		X	X
Group 3 (P–P)	X		X
Group 4 (−-P)			X

This design exceeds the usual possibilities of comparing experimental Group 1 (PTP) with control Group 3 (P–P). By comparing Group 3 (P–P) with Group 4 (−-P), purely practice-and memory-based pretest effects could be estimated (27). Based on insights on how pretesting affects posttest results, a further comparison of experimental Group 2 (-TP) with control Group 4 (−-P) can deliver a clearer picture of the net treatment effects.

### 3.3 Sample description and equivalence check of the compared student groups

The analyses employ data from a longitudinal study conducted as part of the research project EKGe – Extended competence assessment in the healthcare sector, which was funded by the Federal Ministry of Education and Research, Germany. The data were collected between April and July 2021 in 10 nursing schools in North Rhine-Westphalia. In the planning phase of the intervention, the research team contacted all relevant schools by phone and letter. Principals and/or department heads then reported whether at least two parallel classes were available at their respective schools (as experimental and control groups; see section 3.3) and whether teachers were willing to participate with these classes.

Given the frequent alterations to educational practices resulting from the ongoing COVID-19 pandemic, the competence tests were conducted in both remote and in-person formats. To ensure the objectivity of the assessments, they were administered by duly trained personnel in accordance with the instructions delineated in the test manual. The data collection process consisted of two measurement points: the pretest (t_1_) and the posttest (t_2_), with a time gap ranging from 1 to 6 weeks.^2^ Drop-outs from t_1_ to t_2_ totaled *n* = 24 trainees and occurred randomly, for instance due to illness. The final data set includes 332 students who were enrolled in the VET-programs of “geriatric nursing” (*n* = 232), “clinical nursing” (*n* = 82), and “general nursing training” (*n* = 18). The discrepancies in group sizes are attributable to the necessity of employing a convenience sample of classes in the context of ongoing pandemic-related disruptions to the organization of schooling. We assumed that coping with stressful situations is independent of a VET program, and instead dependent only on features of the care situation and its institutional setting. Nevertheless, an examination of the composition of the experimental and control groups with respect to the VET program of their respective members was carried out. Table 2 provides an overview of participant characteristics in each of the four Solomon comparison groups.

**Table 2:** Study groups by training program, year and age group (in percent).

Study group	Training program			Training year			Age group			Total (*N*)
	GER	CLI	GEN	1	2	3	< 21	21–25	> 25	
Group 1 (PTP)	77.8	22.2	0.0	0.0	60.5	39.5	20.3	31.1	48.6	81
Group 2 (-TP)	60.2	21.4	18.4	18.4	67.3	14.3	34.7	40.0	25.3	98
Group 3 (P–P)	65.2	34.8	0.0	0.0	100.0	0.0	45.5	28.8	25.8	69
Group 4 (− -P)	77.4	22.6	0.0	0.0	45.2	54.8	15.8	32.9	51.3	84
Total	69.9	24.7	5.4	5.4	66.9	27.7	28.9	33.8	37.3	332

GER, geriatric nursing; CLI, clinical nursing; GEN, general nursing. Data regarding age group are missing for *n* = 21 trainees.

At the time of the study, most of the students attended their second year of training (*n =* 222). The age of participants is distributed heterogeneously across three age groups. Female trainees dominate the sample (77.9%). Randomized group allocation of the *individual* students was impeded by fixed schooling conditions (class affiliation, school-specific scheduling of theoretical and practical training phases). However, the existing *classes of trainees* were randomly assigned to the four study groups of the Solomon design. To ensure the internal validity of obtained results, the study groups should be comparable in terms of (confounding) factors that may affect their competence acquisition (27). However, an initial examination of Table 2 indicates that group compositions varied with respect to the training program and the year of the classes. A Kruskal–Wallis test revealed that these differences were statistically significant with regard to the training program [*χ^2^*(3) = 13.75; *p* = 0.003]. A Dunn-Bonferroni test further elucidated these differences, specifically identifying a contrast between Group 2 (-TP) versus Group 3 (P–P) (*z* = −3.63; *p* = 0.002). As (33) notes, this finding represents a weak, almost medium effect (*r* = 0.28). Therefore, the students’ training program was controlled for in subsequent multiple regressions together with students’ educational background (see section 4.2). While the *test of stress coping competence* is demonstrated to be non-discriminatory with respect to different training programs in nursing education (13), training affiliation may exert a confounding effect on learning processes during the *intervention phase*.

### 3.4 Test instrument for competence assessment

*Stress coping competence* of nursing trainees was assessed using the *Coping Competence Instrument for Nursing* (CopeCo-N) [see (13), for a detailed description]. This validated instrument provides a Situational Judgment Test embedded in a digital testing environment. Analogous to the instructional unit, the test items are anchored in several short and vivid representations of authentic, prototypical stressful situations that may arise during professional care work in different care settings. A total of nine video vignettes of such challenging scenarios were developed for three fields of practice: *geriatric care*, *inpatient hospital care*, and *ambulatory care*. After watching an introductory video about the work context of a particular setting (e.g., team members, patients/clients, organizational structure), participants see the vignette of a stressful situation in this setting and are prompted to answer test items presented in a multiple-choice format. Only after completing these items (3–4 for each scenario) do they see the next vignette.

Each vignette presents a specific set of stressors, such as: “Being confronted with dying,” “Prioritizing care needs while considering ethical issues,” “Managing multiple care tasks under acute time pressure,” or “Dealing with errors in the care process.” A total of 29 items covers the theoretically derived dimensions of coping competence (see section 2.2). Thus, test takers are first asked to identify the stressors and stress reactions presented in each vignette in order to assess situational demands. They are then asked to select and justify strategic options for coping with these demands in the course and, possibly, in the follow-up to the situation.

The in-depth development process of the Situational Judgment Test involved the collection and synthesis of ratings from experts in nursing science and nursing education, such as experienced nurses and qualified instructors in nursing institutions. They evaluated and optimized all stimuli (i.e., video vignettes) for their descriptive quality and informational content, as well as all items for their technical accuracy and the situational appropriateness of solutions.

A multilevel scoring system serves to rate selected answers. If one of two correct answers was selected, the task was scored with one point. Two points were awarded if both correct answers were selected. Otherwise, zero points were awarded. To investigate whether the pedagogical intervention was effective in terms of competence gains, all 29 test items were used at two measurement points (t_1_ and t*_2_*).

A previous study on test development and validation could establish good construct and criterion validity (13). The theoretically assumed two-dimensional structure (1: Appropriate assessment of the stressful situation; 2: Appropriate selection and justification of coping strategies; see *section 2.2*) exhibits a better fit to the data than a one-dimensional structure. The EAP/PV reliabilities for the partial credit models, which were estimated separately for each of the two measurement points, fall within a good range (*t_1_*: dimension 1 = 0.76 and dimension 2 = 0.85; *t_2_:* dimension 1 = 0.76 and dimension 2 = 0.79). The item characteristics (wMNSQ, *t*-values, discriminatory power) also lie within an acceptable range. In addition, DIF analyses confirmed that the difficulty parameters of the items in all three nursing training programs occupy the same relative position (13).

### 3.5 Hypotheses

The following hypotheses can be derived from the previous considerations and will be subjected to empirical testing:

*H1a*: Trainees in the experimental groups (Group 1, Group 2) demonstrate better performance in the appropriate assessment of stressful situations (dimension 1 of the CopeCo-N) than trainees in the control groups (Group 3, Group 4).

*H1b*: Trainees in the experimental groups (Group 1, Group 2) demonstrate better performance in the appropriate selection and justification of coping strategies (dimension 2 of the CopeCo-N) than trainees in the control groups (Group 3, Group 4).

### 3.6 Data analysis

The statistical examination of the treatment effect consisted of two steps. In the first step, descriptive and bivariate analyses were conducted to assess differences in group-specific test performance. For this purpose, pretest effects are analyzed, and group comparisons are made to estimate treatment-related gains in competence. In a second step, multiple linear regression analyses were conducted to predict criterion variables from several predictor variables. The aim is to examine the influence of the intervention on individual competence levels in the post-test, while accounting for other factors that may influence learning success. Regression models are estimated for the two dimensions of stress management competence separately. The scores achieved in the two test components in the posttest provide the dependent variables. All statistical calculations were performed using SPSS 27 software.

### 3.7 Implementation of the intervention

The instructional unit was implemented by 10 teachers who followed the detailed lesson plans that resulted from previous workshops (see section 2.3). Teachers could contact members of the academic research team at any time if questions arose. They also kept implementation logs to record any deviations from the lesson plans. Overall, there were very few and varied deviations, which were due to unexpected events (e.g., extended lively discussions of certain topics or external interruptions of a lesson). All teachers received identical instructional materials from the research team, i.e., student worksheets, solutions guides, explanatory videos and the radio play/video vignette used for the anchoring situation.

## 4 Results

### 4.1 Descriptive and bivariate results for group comparisons

**Figure 2:**
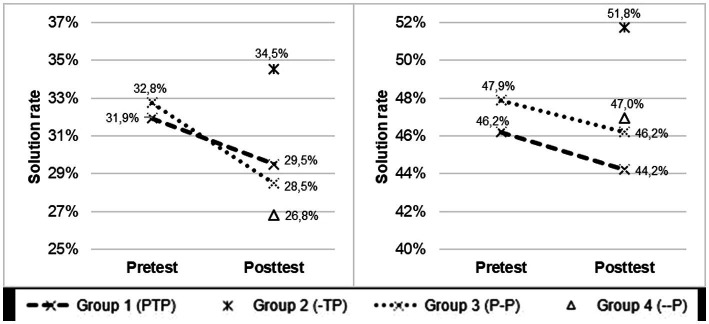
Pre-post comparison of the solution rate in the CopeCo-N for each of the two test components of stress coping skills across the four study groups.

Figure 2 shows the average solution rates achieved in the CopeCo-N in a pre-post comparison, sorted by the tested groups. It is striking that the trainees in experimental Group 2 (-TP), who received the instructional unit but did not have to take a pretest, exhibited the highest solution rates in both test components of the CopeCo-N. Trainees in the additional experimental Group 1 with the additional pretest (PTP), on the other hand, showed a slight decline in their average solution rates for both test components over the two measurement points. Control Group 3 (P–P), which also took the pretest but did not engage in the instructional unit on coping, even exhibits a disproportionately greater drop in performance on the first competence dimension than experimental Group 1 (PTP), which was directly relevant for the comparison. Thus, despite displaying higher levels of prior competence at the beginning of the study (t_1_), Group 3 performed worse on the posttest than Group 1. This counterintuitive decline in test scores can be explained plausibly by decreasing test motivation, which could result from the extensive repeated testing, featuring complex situational judgment tests with several stress situations, and only a short time interval between pretest and posttest (ranging from 1 to 6 weeks). This interpretation is supported by a marked decrease in the average amount of processing time invested by the participants when taking the tests, depending on the time of measurement in both the experimental Group 1 (PTP) and the control Group 3 (P–P). While the processing time in the pretest (t_1_) averaged 42.3 min, the time spent on the posttest (t_2_) was only 35.9 min on average (− 6.4 min). It should therefore be noted that participants did not work on the test items of the CopeCo-N with the same intensity in the pretest (t_1_) and the posttest (t_2_). Although the shorter time taken to complete the test could also be due to familiarity with the test items, the marked decline in test scores suggests that memory and practice effects are less likely.

Given the declining test scores and time spent on repeated measures for all participants who received a pre-and post-test, indicating fatigue effects rather than practice/memory effects, further analyses focus on comparing experimental Group 2 (-TP) with control Group 4 (−-P) to estimate the pure treatment effects (*cf. section 3.2*). A t-test for independent samples shows that these two groups differ significantly in terms of their scores on the first component of the posttest (t_2_) *(i.e., the appropriate assessment of stressful situations)*. This effect is close to a medium size (32) [*t*(180) = 3.07; *p* = 0.002; *d* = 0.46]. A comparison of test performance in the second component *(*i.e.*, in the appropriate selection and justification of coping strategies)* further shows that experimental Group 2 and control Group 4 also differ nominally by approximately 5% in their solution rates, although this difference is not statistically significant. These treatment-related differences in test performance will therefore be examined in more detail while controlling for other potentially confounding factors at the observational level of individual participants.

**Figure 3:**
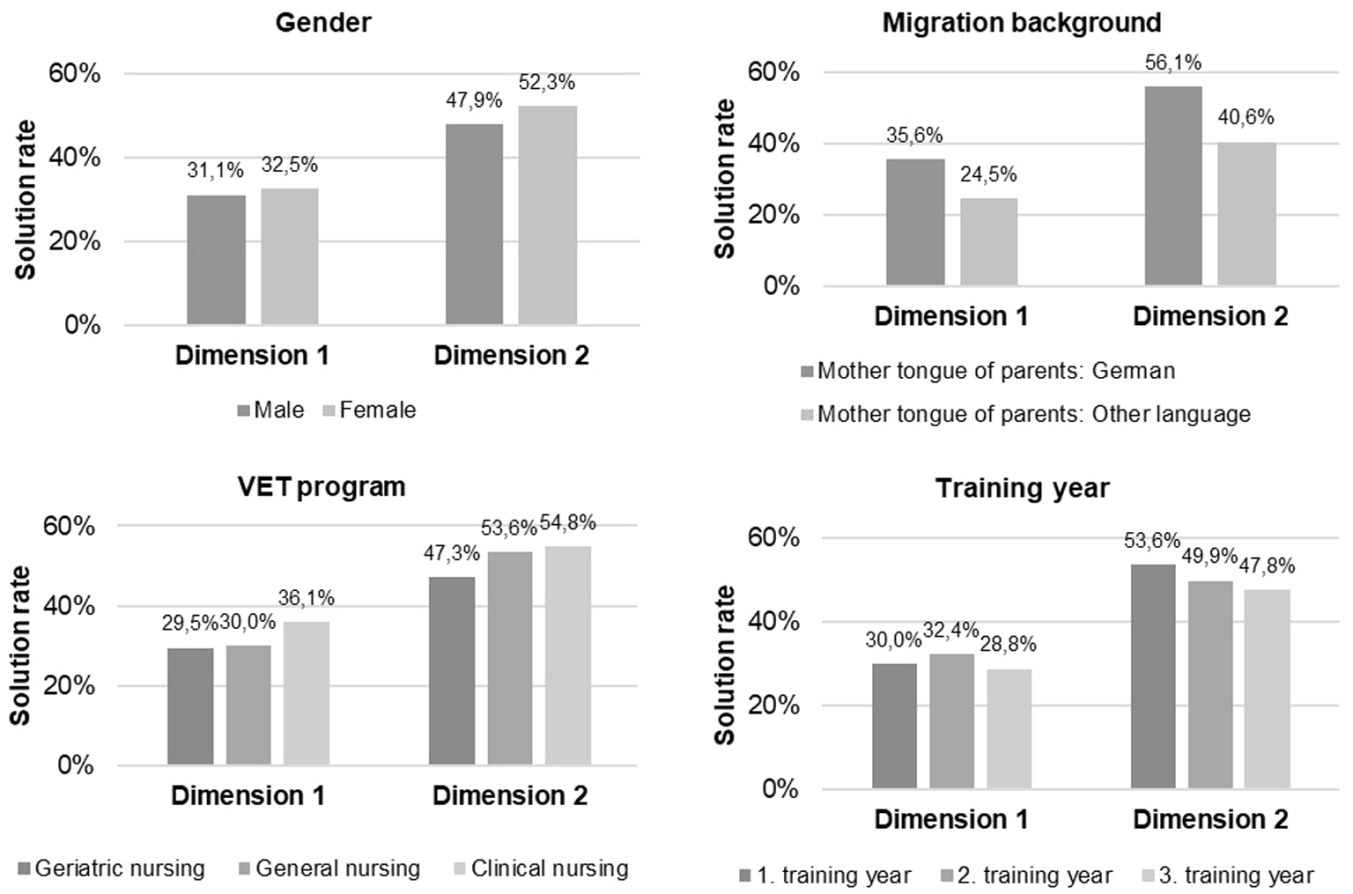
Test results in the CopeCo-N in the posttest (t2) for each of the two dimensions of stress coping competence according to potentially confounding factors of competence gains (shown as percentages).

As a first insight into the confounding factors of individual competence gains, Figure 3 depicts average solution rates in the CopeCo-N for experimental Group 2 (-TP) and control Group 4 (−-P) as a function of individual student characteristics. It is noticeable that solution rates are higher in the second test component *(selection and justification of coping strategies)* than in the first *(assessment of stressful situations).*^3^ Furthermore, the average solution rates vary considerably depending on the immigrant background of the test taker, as indicated by the native language of both parents. Significant differences are also attributable to the formal training program, as trainees in the clinical nursing program outperform trainees in the geriatric nursing program. Although experimental Group 2 and control Group 4 display similar proportions of members from different programs (see section 3.3), individual membership in a particular program is controlled for in the next step of estimating the treatment effect. Students’ immigrant background is also included in the analysis.

### 4.2 Results of linear regressions at the individual level

Table 3 shows the results of linear regression analysis used to predict the two dimensions of stress coping competence at the time of the posttest for trainees in the experimental Group 2 (-TP) and the control Group 4 (−-P).^4^ Model 1 specifies the intervention effect on the appropriate assessment of stressful situations (dimension 1) while estimating influences of training program and migration background on test performance, simultaneously. The results confirm the previous finding from the t-test for independent samples and support the assumption that participation in the newly developed instructional unit has a significantly positive effect on the *appropriate assessment of stressful situations* (*β* = 0.212, *p* = 0.006). Moreover, the participants’ migration background proves to be the strongest predictor of this test component (*β* = −0.281, *p* < 0.001). However, the specific training program for nursing in which a test participant is enrolled in, lacks a significant effect when controlling for immigrant background.

**Table 3:** Linear regression analyses to explain test scores of Group 2 (-TP) and Group 4 (−-P) in the CopeCo-N, sorted by the two test components.

	Model 1 (1) Appropriate assessment of the stressful situation			Model 2 (2) Appropriate selection and successful justification of coping strategies		
	B	β	SE	B	β	SE
(Constant)	9.310		0.646	15.107		0.646
Group membership (ref. no treatment)						
Participation in the intervention	2.130	0.212***	0.760	0.490	0.048	0.759
Training program (ref. geriatric nursing)						
General nursing	−1.263	−0.077	1.250	1.063	0.064	1.250
Clinical nursing	1.011	0.085	0.872	0.897	0.074	0.872
Mother tongue of parents (ref. German)						
Other language	−3.028	−0.281***	0.778	−4.269	−0.391***	0.778
Corrected R^2^	0,125			0.151		
N	172			172		

***p < 0.01, **p < 0.05, *p < 0.10; B = unstandardized regression coefficients, β = standardized regression coefficients, SE = standard error.

Similarly, model 2 shows that the test takers’ migration background significantly (negatively) affects the *appropriate selection and successful justification of coping strategies* (*β* = −0.391, *p* = 0.039) while the formal nursing training program does not. In contrast, participation in the newly developed instructional unit is positively related to this particular competence dimension but loses significance after controlling for the confounding factors of individual learning gains.

## 5 Discussion

### 5.1 Main findings

Consistent with the research hypotheses, group comparisons and regression models revealed that participants in a targeted pedagogical intervention at nursing schools outperform other study groups on a validated Situational Judgment Test that assesses the *stress coping competence* of novice nurses. This applies under the condition that no repetition effects of previous testing are present and that confounding factors relevant to learning, such as enrollment in a specific training program (for geriatric, clinical, or general nursing), are simultaneously controlled for. The newly developed instructional unit for acquiring *stress coping competence* has a markedly positive effect on the students’ abilities of *assessing stressful situations*, which presents the first dimension of the competence model. Therefore, the instructional unit demonstrably contributes to a differentiated reflection and evaluation of these situations. Considering the theoretical foundations of the intervention, a nuanced understanding of extant stressors and impending stress reactions provides the indispensable basis for *coping flexibility*. Complementing this picture, the experimental group also demonstrated better, although not statistically significant, test performance in the second dimension of *stress coping competence*, which measures the adequate *selection and justification of coping strategies* for each stressful situation. Taken together, these results indicate that our intervention, aiming at heightened awareness of multiple stressors in the nursing profession as well as varied and reasonable ways to deal with these stressors, rather than promoting and routinizing a singular, presumably superior coping *style*, achieved its intended objectives. The fact that observable gains in the second dimension of *stress coping competence* are less pronounced than in the first dimension could be due to the placement of the posttest within the study design. The final assessment with the test instrument CopeCo-N was scheduled close to the end of the intervention, thus eliminating a subsequent practical training phase at a care facility. As a result, participants missed opportunities to transfer coping strategies into their work practice. Thus, they did not (yet) gain practical experience in executing coping strategies that could enrich their strategic reasoning in authentic test situations. On the other hand, developing *coping flexibility* (and perhaps even overcoming rigid coping styles that may have already become a habit) is a longer process that requires repeated reflection and refinement of strategy selection and execution.

A look at a recent meta-analysis on the effectiveness of stress management interventions [specifically in hospitals; (9)] shows that the design and results of the newly developed instructional unit for prospective nurses complements the scientific evidence on the target group of experienced registered nurses. Regarding the level of intervention, a person-directed approach yielded larger effects than organization-directed and multilevel approaches. As with the instructional unit evaluated in the present study, person-directed interventions seek to enhance employees’ abilities to cope with stressors and regulate stress responses, rather than modifying the contextual causes of stress by reducing job demands and/or enhancing job resources within the nursing setting. Nevertheless, the superiority of the person-directed approach could only be established for its short-term effect on experienced levels of stress, as the majority of available studies lack follow-up measurements beyond 6 months post-intervention.

In regard to potential moderators, the meta-analysis found that heightened exposure correlates with enhanced efficacy, i.e., for interventions in which the study sample participated in the majority of the scheduled sessions. Consequently, the authors advocate for the implementation of targeted measures to enhance adherence and facilitate the transfer of learning. This argument lends support to our proposed explanation of the comparably small competence gains observed in the second component of the investigated stress coping competence, namely the selection and justification of adequate coping strategies. As the authors elucidate, the acquisition and retention of new skills necessitate shifts in cognitive processes and professional actions. If newly acquired skills are not practiced regularly and integrated into one’s daily routine, they may lose their efficacy over time.

Finally, given that the majority of available effectiveness studies employ indicators of stress and strain as outcome variables, the incorporation of an assessment of *stress coping competence* can be a valuable extension. It can be reasonably assumed that demonstrable gains in competence, that is, the participants’ internal prerequisites for situational judgments as well as strategic choices, may be an important mediating factor in the reduction of stress and strain experienced and reported later on at the workplace.

Another finding of our intervention study deserves attention both from a research perspective and a practical perspective on the professional development of prospective nurses. Moreover, it corresponds with the meta-analytic results from de Wijn and van der Doef (9), indicating that the effectiveness of interventions increases with the homogeneity of the trained group of nurses. The present study revealed differing average solution rates in the CopeCo-N between students with and without a migration background (indicated by the mother tongue of their parents). In this Situational Judgment Test, some learner groups might have experienced a language barrier, which caused difficulties in reading and interpreting test instructions or items correctly.

A similar outcome has been observed in previous studies within the field of healthcare vocations. At the conclusion of their vocational training, medical assistance trainees with a non-German native language demonstrated a markedly inferior performance in the professional skills assessment relative to their peers with a German native language. Specifically, reading skills had a significant impact on test results (28). Differentiated insights stem from a panel study across diverse occupational categories (29). The results of this study also indicate that successful trainees with a migration background were less prone to receive a favorable grade in the concluding examination relative to non-migrants. Furthermore, these students exhibited a diminished likelihood of transitioning into qualified employment. However, after controlling for other potential influencing factors, including school degree, grades in the school graduation certificate, family background, age, and training in the desired occupation, no significant effects of a migration background on the various success indicators could be observed. These indicators pertain to successful vocational degree, grades of “good” or “very good” in the final examination, and qualified employment. Taken together, these preceding, domain-specific studies indicate that ethnic inequalities among vocational trainees are not necessarily intensified. However, there is also no compensation for disadvantages in educational attainment that occur due to social background and previously acquired education.

### 5.2 Limitations and research perspectives

While the reported study yielded positive findings, it is not without shortcomings. This paper addresses four noteworthy aspects, along with suggestions for methodological extensions to the design and instruments of future intervention studies.

#### Low generalizability of findings

The reported intervention was carried out in a German federal state, and the instructional unit was planned according to the didactic principles of the German vocational training system. Under the difficult conditions of the COVID pandemic, classes from each of the three parallel nursing programs (geriatric, clinical, and general nursing) had to be recruited as convenience samples. In addition, the Ministry of Education restricted the collection of data on several individual characteristics of the students. As a result, very few individual prerequisites for successful learning could be included in the statistical analyses. For example, there is no individual information on school qualifications prior to entering nursing training, which may well be relevant in explaining the acquisition of skills at a nursing school. Thus, replication studies with representative samples for the VET system are needed.

#### Lack of long-term follow-up

Our study focuses primarily on pre-and post-intervention competence gains. As a result, we cannot determine whether the newly developed instructional unit remains effective over time or whether the participating students can effectively transfer the acquired coping strategies into their actual nursing practice. Therefore, and especially with regard to the second dimension of stress coping competence, a follow-up survey to capture the medium-or long-term effects of the intervention seems to be an insightful, but not yet applied, extension of the study design. This extended design should definitely include a thorough examination of factors in care institutions that facilitate or hinder the transfer of learning from the school to the work context.

#### Competency assessment based on authentic scenarios cannot fully substitute for observation and is potentially tiring

The video-simulated Situational Judgment Test, while innovative and relevant, may not fully capture students’ ability to cope with real-life nursing situations. Actual work performance may be influenced by many other factors (e.g., teamwork) that cannot be fully replicated in a controlled environment. This is particularly true in the extraordinary working conditions that existed during the COVID pandemic. In addition, the elaborate and time-consuming nature of the test appears to have led to test fatigue effects in the two groups that underwent pre-and post-intervention competency assessment. Future studies should therefore investigate whether shorter versions of the CopeCo-N can be used to estimate individual competence levels without compromising diagnostic accuracy. Another interesting option would be to conduct competency testing in skills labs. These labs are not only fully equipped to simulate professional nursing work in typical care settings and allow observation of the decisions and actions taken by student nurses. They also provide space for debriefing and feedback to encourage reflection and re-engagement with a particular scenario. Thus, skills labs can have the dual benefit of providing an observation-based assessment of acquired skills and providing additional learning opportunities after testing.

#### The unresolved role of potential language barriers among test takers

Our results point to disadvantages for students with an immigrant background. A further optimization of the test instrument would include a statistical examination of the language sensitivity of all items, but also the development of test versions in different languages. With regard to the instructional phase of the intervention, further development should focus on differentiated didactic support for different groups of learners. This may include multiple resources to present key learning content in alternate ways.

Despite these limitations, the presented instructional unit to build and strengthen *stress coping competences* among future nurses together with a validated and authentic test instrument for assessing these competences (13) provide a fruitful ground for future research on the professional development in an important vocational sector.

## Data Availability

The datasets presented in this study can be found in online repositories. The names of the repository/repositories and accession number(s) can be found at: https://www.iqb.hu-berlin.de/fdz/studies/EKGe.

## Footnotes

^1^For detailed insights into the design of the instructional unit and the evaluation with regard to other target variables, please refer to Vorpahl (34).

^2^The varying distance between the first and second measurement points was due to the different successions and lengths of theoretical training phases (i.e., lessons in schools) and practical training phases (i.e., in care settings such as hospitals) for the participating classes. Only trainees who took the test at *both* measurement points in experimental Group 1 (PTP) and control Group 3 (P–P) were included in the analyses.

^3^The discrepancies between the two test components may be due to the fact that the Situational Assessment consists of the appropriate identification of stressors and stress reactions depicted in a scenario. Stress reactions describe the psychological or psychosomatic impact of stressors on an individual (e.g., feelings of despair). Test takers may have had more difficulty selecting the correct descriptions of these potential experiential qualities of stressful encounters than in their strategic selection and reasoning for dealing with the situational demands.

^4^Application requirements for the regression analysis were checked for both models. High tolerance values indicate that there is no multicollinearity in either model. The residuals are normally distributed. The non-significant results of the Breusch-Pagan test indicate the absence of heteroscedasticity.
